# Invited Commentary: Detecting Individual and Global Horizontal Pleiotropy in Mendelian Randomization—A Job for the Humble Heterogeneity Statistic?

**DOI:** 10.1093/aje/kwy185

**Published:** 2018-09-05

**Authors:** Jack Bowden, Gibran Hemani, George Davey Smith

**Affiliations:** MRC Integrative Epidemiology Unit, University of Bristol, Bristol, United Kingdom

**Keywords:** heterogeneity statistic, horizontal pleiotropy, Mendelian randomization, MR-Egger regression, outlier detection

## Abstract

Mendelian randomization (MR) is gaining in recognition and popularity as a method for strengthening causal inference in epidemiology by utilizing genetic variants as instrumental variables. Concurrently with the explosion in empirical MR studies, there has been the steady production of new approaches for MR analysis. The recently proposed “global and individual tests for direct effects” (GLIDE) approach fits into a family of methods that aim to detect horizontal pleiotropy—at the individual single nucleotide polymorphism level and at the global level—and to adjust the analysis by removing outlying single nucleotide polymorphisms. In this commentary, we explain how existing methods can (and indeed are) being used to detect pleiotropy at the individual and global levels, although not explicitly using this terminology. By doing so, we show that the true comparator for GLIDE is not MR-Egger regression (as Dai et al., the authors of the accompanying article (*Am J Epidemiol*. 2018;187(12):2672–2680), claim) but rather the humble heterogeneity statistic.

Mendelian randomization (MR) (1) is gaining in recognition and popularity as a method for strengthening causal inference in epidemiology by utilizing genetic variants as instrumental variables. Its use has been accelerated in recent years by the increasing availability of genome-wide association studies and large-scale biobank cohort data. Indeed, most traits that are analyzed with sufficiently large sample sizes (e.g., hundreds of thousands of individuals) yield large numbers of robustly associated variants. Human height is perhaps the most extreme example, with over 3,000 independent variants identified so far (2). To ascertain, for example, whether height exerts a generic causal effect on risk of colorectal cancer, we would need to assume that each variant additionally 1) is not associated with any confounders of the height–colorectal cancer relationship and 2) only affects the risk of colorectal cancer through height (or an automatic concomitant of height, such as cell number). It seems implausible in this case that all 3,000 variants will meet these strict assumptions, due to their exerting an influence on multiple downstream traits through many different pathways. This phenomenon is referred to as *horizontal pleiotropy* (3, 4), and its existence is well documented (5).

Concurrently with the explosion in empirical MR studies, there has been the steady production of new approaches for MR analysis. For example, simple methods have been adapted from mainstream meta-analysis to synthesize causal estimates obtained from many independent variants, while accounting for both heterogeneity and bias due to pleiotropy. These include the inverse-variance–weighted (IVW) approach (6), MR-Egger regression (7) and multivariate extensions thereof (8, 9). Another stream of methods instead aim for natural robustness to pleiotropy, rather than enacting an explicit bias correction. These include the weighted median estimator (10) and mode-based estimation (11, 12). (For a recent review, see Hemani et al. (13).) All of these methods require only summary data estimates of single nucleotide polymorphism (SNP)–trait associations that are often nondisclosive and publicly available (14).

The approach recently proposed by Dai et al. (15), termed “global and individual tests for direct effects” (GLIDE), fits into a family of methods that aim to detect horizontal pleiotropy—at the individual SNP level and at the global level—and to adjust the analysis by removing outlying SNPs. Specifically, the GLIDE method is introduced for contexts in which individual-level data are available on a set of genetic instruments, an exposure and a binary outcome, and where the data have been collected under case-control sampling. A relative risk model is used under a rare disease assumption to address the issue of noncollapsibility, and inverse probability weighting is used to adjust for ascertainment bias. It uses *P* values derived by simulation and a *P* value combination approach to derive tests for pleiotropy. Dai et al. show that the GLIDE method is far more powerful at detecting global pleiotropy than is MR-Egger regression (15).

Verbanck et al. (5) have also recently proposed a new test for global pleiotropy based on a simulated unweighted heterogeneity statistic, as well as an approach with which to detect and remove individual outliers from the analysis. Their method—“Mendelian randomization pleiotropy residual sum and outlier” (MR-PRESSO) is close in spirit to GLIDE.

It is welcome that the MR problem is now being scrutinized by many independent scientific groups across the world. This will undoubtedly lead to improved methods, inference, and understanding. In this commentary, we explain how existing methods (partly borrowed from mainstream meta-analysis) can, and indeed are, being used to detect pleiotropy at the individual and global levels, although not explicitly using this terminology. By doing so, we show that the true comparator for GLIDE is not MR-Egger regression but rather the humble heterogeneity statistic.

## COCHRAN’S *Q* STATISTIC

Following equation 2 in the paper by Dai et al. (15) and using their notation, we start by assuming the causal relative risk model for the outcome Y given the jth genetic variant (or SNP) $G_{j}$ out of m holds in a cohort (e.g., cohort 1) of individuals

(1) $$\log\{\mathrm{Pr}(Y=1|G_{j})\}=\beta_{0}^{⁎}+(\beta_{1}\alpha_{j}+\beta_{2j})G_{j}.$$

**Figure 1.**
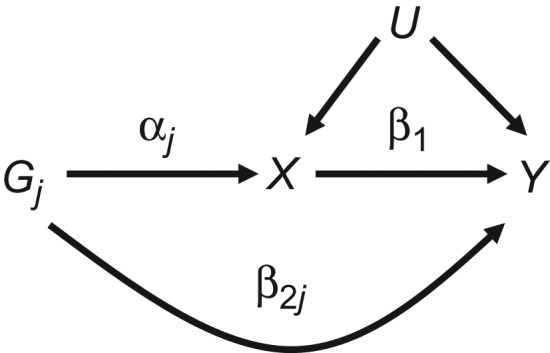
Relationship between genetic variant *G*_*j*_, exposure *X*, outcome *Y*, and unmeasured variable(s) *U*, with effects as defined in equation 1.

Here $\alpha_{j}$ represents the association between the *j*th SNP and the exposure *X*, $\beta_{2j}$ represents the pleiotropic effect of SNP *j* on the outcome *Y*, and $\beta_{1}$ represents the causal effect of *X* on *Y* we wish to estimate. This setup is illustrated in Figure 1, where we assume that horizontal pleiotropy operates via pathways that are independent of the exposure, although this is not crucial to any of our following arguments.

Assume, as Dai et al. do (15), that an independent, external data source (e.g., cohort 2) is available with which we can obtain an estimate for the parameter $\alpha_{j}$ with negligible error. That is, our estimate ${\hat{\alpha }}_{j}\approx \alpha_{j}$. This requires cohort 2 to be large and also homogenous with respect to cohort 1 (16, 17). Further assume that our estimate for the jth SNP-outcome association has variance $\sigma_{Yj}^{2}$. We can then derive an estimate, ${\hat{\beta }}_{j}$, for the causal effect parameter $\beta_{1}$. This is obtained by dividing the jth SNP-outcome association ${\hat{\beta }}_{YGj}$ by the *j*th SNP-exposure association $\alpha_{j}$. The estimate ${\hat{\beta }}_{j}$ has mean and variance

$$\begin{array}{c} \;E\left[{\hat{\beta }}_{j}\right]=\frac{1}{\alpha_{j}}E\left[{\hat{\beta }}_{YGj}\right]=\beta_{1}+\frac{\beta_{2j}}{\alpha_{j}},\\ \mathrm{Var}\left[{\hat{\beta }}_{j}\right]=\frac{1}{\alpha_{j}^{2}}\mathrm{Var}\left[{\hat{\beta }}_{YGj}\right]=1/w_{j}=\frac{\sigma_{Yj}^{2}}{\alpha_{j}^{2}}. \end{array}$$

The inverse-variance–weighted average of the m ratio estimates ${\hat{\beta }}_{\mathrm{IVW}}=\underset{j}{\sum }w_{j}{\hat{\beta }}_{j}/\sum w_{j}$. Finally, define Cochran’s Q statistic $Q=\underset{j}{\sum }Q_{j}=\underset{j}{\sum }w_{j}{\left({\hat{\beta }}_{j}-{\hat{\beta }}_{\mathrm{IVW}}\right)}^{2}$. When all m SNPs are uncorrelated, and the independent samples in cohort 1 and cohort 2 are drawn from the same underlying population, the IVW estimate is asymptotically equivalent to the 2-stage least-squares estimate that would be obtained with individual-level data. If $\beta_{2j}=0$ for all *j* in (1, …, *m*), so that the global null hypothesis of no pleiotropy is true, then

- ${\hat{\beta }}_{\mathrm{IVW}}$ is an unbiased estimate for $\beta_{1}$;
- Cochran’s *Q* statistic should follow a χ^2^ distribution with m−1 degrees of freedom (df); and
- provided that m is sufficiently large, the jth contribution to Q, $Q_{j}$, is approximately χ^2^ distributed on 1 df.

Therefore, Q can be used to test for global pleiotropy and $Q_{j}$ can be used to test for individual pleiotropy. The use of Cochran’s Q statistic makes perfect sense, since it is equivalent to the Sargan test statistic for detecting invalid instruments (18) from the econometrics literature.

## MR-EGGER REGRESSION

When $\beta_{2j}\neq 0$ for some j in 1, …, *m*, then the IVW estimate can still unbiasedly estimate the causal effect when 1) the sample covariance between $\alpha_{j}$ and $\beta_{2j}$ is zero—the “instrument strength independent of direct effect” (InSIDE) assumption—and 2) the sample mean of the $\beta_{2j}$ terms is zero. This is referred to as *balanced pleiotropy*. Pleiotropy is said to be “directional” if the InSIDE assumption holds but the sample mean of the $\beta_{2j}$ terms is nonzero. The MR-Egger method performs a regression of the SNP-outcome associations on the SNP-exposure associations with the intercept left unconstrained to test this hypothesis, by assuming the mean model:

$$E\left[{\hat{\beta }}_{YGj}\right]=\beta_{0E}+\beta_{1E}\alpha_{j}.$$

The MR-Egger method still relies on the InSIDE assumption, but if satisfied the intercept term $\beta_{0E}$ provides an estimate of the mean pleiotropic effect, and the slope $\beta_{1E}$ provides an estimate of the causal effect $\beta_{1}$ adjusted for any nonzero mean pleiotropy. After fitting the MR-Egger regression model and adjusting for the mean pleiotropic effect, it is then possible to test whether any residual heterogeneity due to pleiotropy remains. This can be assessed by using Rucker’s *Q* statistic:

$$Q'=\underset{j}{\sum }Q_{j}^{'}=\underset{j}{\sum }w_{j}{\left({\hat{\beta }}_{j}-\frac{{\hat{\beta }}_{0E}}{\alpha_{j}}-{\hat{\beta }}_{1E}\right)}^{2}.$$

Under the null hypothesis that all SNPs have the same direct effect ${(\beta }_{2j}=\beta_{2})$, estimating and adjusting for their mean value (via ${\hat{\beta }}_{0E}$) is sufficient to completely remove all pleiotropy from the analysis. If this is true (which is unlikely):

- Rucker’s Q′ statistic should follow a χ^2^ distribution with m−2 df and
- The *j*th component of Q′, $Q_{j}^{'}$ should approximately follow a χ^2^ distribution on 1 df.

Rejection of the null hypothesis implies that residual direct effects, with magnitudes $\beta_{2j}-{\hat{\beta }}_{0E}$, remain in the data. Therefore, Rucker’s Q′ statistic and its individual components $Q_{j}^{'}$ can be used to test for global and individual pleiotropy after MR-Egger adjustment.

## SIMULATION EXAMPLE

**Figure 2.**
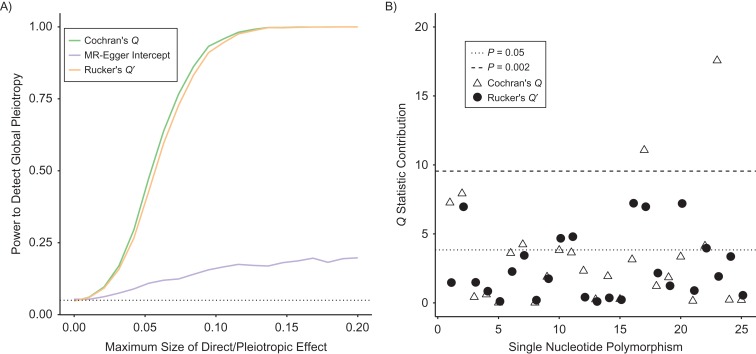
A) Power of Cochran’s *Q* statistic, Rucker’s *Q*′ statistic, and the Mendelian randomization (MR)-Egger intercept to detect global pleiotropy for simulated MR data containing 25 single nucleotide polymorphisms. B) Individual contributions to Cochran’s *Q* statistic and Rucker’s *Q*′ statistic. The single nucleotide polymorphisms were individually numbered from 1 to 25 for illustrative purposes. Horizontal lines indicate the 95th (dotted lines) and 99.8th (dashed line) percentiles of the χ^2^ distribution with 1 degree of freedom.

To illustrate the above, Figure 2A shows the power of Cochran’s Q statistic and Rucker’s Q′ statistic to detect global pleiotropy at the 5% significance level for simulated MR data sets of 25 SNPs. Also shown on the same plot is the power of the MR-Egger method to detect a statistically significant intercept at the 5% level. The simulation assumes balanced pleiotropy, but we vary the magnitude of the pleiotropic effects so that their maximum value lies between 0 and ±0.2. The simulation is therefore analogous to Figure 2B in the paper by Dai et al. (15).

The power of Cochran’s Q statistic and Rucker’s Q′ statistic to detect global pleiotropy is seen to increase sharply as a function of direct effect magnitudes, whereas the power of MR-Egger to detect a nonzero intercept increases slowly. In fact, its power depends strongly on the amount of variability in the instrument strengths across the set of SNPs (17, 19). The key message we wish to convey in this commentary is the following:

*MR-Egger regression provides a very poor test for global pleiotropy, and it was never intended to be used for this purpose. Cochran’s*Q*statistic and Rucker’s*Q′*statistic should instead be used.*

Figure 2B shows, for a single MR analysis containing 25 SNPs, the individual contributions to Cochran’s Q statistic and Rucker’s Q′ statistic. This time, the data are generated under directional pleiotropy with mean value 0.1 to induce a difference between the Q and Q′ contributions. Horizontal lines indicate the 95th percentile and Bonferroni-corrected 99.8th percentile of the χ^2^ (1 df) distribution; the latter threshold can be used to guide the detection of individual outliers in order to control the familywise error rate. In this case, 2 variants’ contributions to Q are extreme enough to be considered for removal. The fact that no variant’s contribution to Q′ is large enough to warrant removal is a sign that the MR-Egger model constitutes a better fit to the data than the IVW model in this instance. This is not surprising because it is a 2-parameter rather than a 1-parameter model. The R computer code (R Foundation for Statistical Computing, Vienna, Austria) used to perform the simulations in Figure 2 can be found in the accompanying Web Appendix (available at https://academic.oup.com/aje).

A framework for using Q and Q′ to detect global pleiotropy and decide on the appropriateness of either IVW or MR-Egger regression for a given analysis is contained in an article by Bowden et al. (17). (See also Thompson et al. (20) and Schmidt and Dudbridge (21) for closely related Bayesian approaches.) The GLIDE method proposed by Dai et al. (15) is an interesting addition to the literature on MR methods, but it should be compared directly with Cochran’s Q statistic and Rucker’s Q′ statistic when evaluating its utility in this regard. It may well give very similar results.

Because of their derivation under the 2-stage least-squares framework, the IVW and MR-Egger approaches are only approximate when the outcome is binary, due to the noncollapsibility of the odds ratio (22), and case-control sampling is used. The GLIDE approach does at least address these issues head-on with the use of a causal relative risk model. However, when the binary outcome has a relatively low prevalence, a more straightforward logistic regression with inverse probability weighting provides a convenient and reasonable way to obtain parameter estimates for GLIDE’s causal relative risk model (23). Indeed, in the authors’ own applied example, this is the approach taken (15). Furthermore, because each SNP explains only a small amount of variation in the exposure in an MR study and makes up a small contribution of the total instrument strength, a linear model provides a surprisingly accurate approximation with which to identify a “local” causal effect. For further details of this argument, see Appendix A.2 in another paper by Zhao et al. (24).

## COMPARISON WITH MR-PRESSO AND THE MR ROBUST ADJUSTED PROFILE SCORE

Dai et al. assume in the development of their method that SNP-exposure associations utilized in the analysis are estimated with negligible error (i.e., that they are strong instruments) (15). This is referred to as the “no measurement error” assumption (25). When the no measurement error assumption is violated due to the presence of weak instruments, the standard meta-analytical framework outlined in this commentary breaks down and the type I error rate of Cochran’s Q statistic for detecting pleiotropy can be grossly inflated. This fact has also been noted by Verbanck et al. (5), which provided the motivation for MR-PRESSO. In recent work (25), we modified the inverse variance weights used to calculate Cochran’s Q statistic to improve their performance with weak instruments. Verbanck et al. helpfully compared MR-PRESSO with a preliminary version of our modified Q statistic and showed that it performs similarly (5).

Both MR-PRESSO and GLIDE use tests for individual pleiotropy to explicitly remove variants from the analysis before estimating the causal effect. A simpler and arguably more objective approach is to use robust estimators that penalize, rather than remove the contribution of, outlying variants. See, for example, the MR “robust adjusted profile score” approach of Zhao et al. (24), which also accounts for bias due to weak instruments.

In conclusion, we hope we have explained how to use the standard heterogeneity statistic to learn about individual and global pleiotropy in MR, as a useful comparator to GLIDE. Further comparison of all of these approaches is needed to reach a better understanding of their relative merits.

## Supplementary Material

Web Material

## Abbreviations

df: degrees of freedom
GLIDE: global and individual tests for direct effects
InSIDE: instrument strength independent of direct effect
IVW: inverse-variance–weighted
MR: Mendelian randomization
MR-PRESSO: Mendelian randomization pleiotropy residual sum and outlier
SNP: single nucleotide polymorphism
